# Video laryngoscopy in neonate and infant intubation—a systematic review and meta-analysis

**DOI:** 10.1007/s00431-024-05839-2

**Published:** 2024-11-20

**Authors:** Ilari Kuitunen, Kati Räsänen, Tuomas T. Huttunen

**Affiliations:** 1 Institute of Clinical Medicine, University of Eastern Finland, Kuopio, Finland; 2 Department of Pediatrics and Neonatology, Kuopio University Hospital, Puijonlaaksontie 2, Kuopio, Finland; 3 Department of Cardiothoracic Anesthesia, Tampere Heart Hospital, Tampere, Finland; 4 Faculty of Medicine and Health Technologies, Tampere University, Tampere, Finland

**Keywords:** Intubation, Neonatal intensive care, Pediatric intensive care, Airway management

## Abstract

We aimed to analyze the effect of video laryngoscopy on intubation success, time to intubation, and adverse events in infants and neonates. A systematic review and meta-analysis was performed, for which a neonates (age less than 29 days) and infants (age less than 365 days) needing to be intubated were included. The main outcomes were first attempt success rate in the intubation, time to intubation, and adverse events. Evidence certainty was assessed according to GRADE. We included 13 studies. Seven studies with 897 patients focused on neonates, and the first attempt success rate was higher in the video laryngoscopy group (RR 1.18, CI: 1.03–1.36). Six studies included 1039 infants, and the success rate was higher in the video laryngoscopy group (RR 1.06, CI: 1.00–1.20). Time to intubation was assessed in 11 trials, and there was no difference between the groups (mean difference 1.2 s, CI − 2.2 s to + 4.6 s). Odds of desaturation (OR 0.62, CI 0.42–0.93) and nasal/oral trauma (OR 0.24, CI 0.07–0.85) were lower in the video laryngoscopy group. Evidence certainties varied between moderate and low.

*Conclusion*: We found moderate certainty evidence that the use of video laryngoscopy improves first attempt success rates in neonate and infant intubations, while the time to intubation did not differ between video and direct laryngoscopy groups. Further studies are still needed to improve the first intubation success rates in neonates.

**Table:** 

**What is known:** *• Video laryngoscopy has been shown to improve first-pass intubation success rates and reduce time to intubation in adults and older children.* **What is new:** *• Video laryngoscopy improved the first attempt intubation success rates both in neonates and in infants.* *• Video laryngoscopy did not increase the time to intubation, and it was associated with less adverse events than direct laryngoscopy.*

**Supplementary Information:**

The online version contains supplementary material available at 10.1007/s00431-024-05839-2.

## Introduction

Video laryngoscopy has been proven to improve intubation success in adults leading to its widespread adoption in clinical practice [1–3]. However, its use and effectiveness in infant and neonatal intubation remain a topic of debate [4]. A previous Cochrane review concluded that video laryngoscopy would not improve intubation success rates in older children; however, this review excluded neonates and infants [5]. A previous Cochrane review reported that video laryngoscopy may improve intubation success rates in neonates, but it did not include infants [6]. Some studies have shown improved success rates with video laryngoscopy, while some have not found evidence of a difference [7–9]. Most recently European Society of Anaesthesiology and Intensive Care and British Journal of Anaesthesia joint guidelines gave a strong recommendation based on moderate evidence certainty to use video laryngoscopy in infants and neonates [10].

Intubation indications for neonates differ notably, as these intubations are mostly done due to respiratory issues in the early phase and are performed in an acute setting [11–14]. In older infants and children, the majority of intubations are performed in a controlled operation room environment before surgery [15]. As previous reviews have excluded infants, and the evidence regarding the intubations in neonates is so far sparse, it is essential to investigate the use of video laryngoscopy specifically in these patients to determine its effectiveness and potential benefits.

By design, video laryngoscopy should provide a (better) visualization of the upper airway anatomy, assisting the healthcare provider in achieving a successful tracheal intubation. As the number of neonates needing intubation has fallen, the experience and skill of airway management among pediatricians and neonatologists has declined. This shift is due to changes in the early management of neonates [16].

Thus, this systematic review and meta-analysis aimed to analyze the effectiveness of video laryngoscopy on intubation success and adverse events compared to direct laryngoscopy in neonates and infants.

## Methods

### Study design

We performed a systematic review and meta-analysis of randomized controlled trials.

### Search process

We searched Pubmed, SCOPUS, and Web of Science databases initially on October 31, 2023, and updated the search on May 31, 2024. The complete search strategy is provided in the supplementary materials. The results were uploaded to Covidence software for screening. Two authors screened each abstract and full text independently, and cases of disagreement were solved by a mutual consensus. We did not search grey literature such as conference abstracts or other non-peer-reviewed publications and databases. We hand searched the reference lists of the included articles for potential additional articles suitable to be included. We decided to include only English published results, as the databases we searched contain mostly studies published in English.

### Inclusion and exclusion criteria

We used the following inclusion criteria. Patients were neonates or infants requiring endotracheal intubation. A neonate was classified as a child aged less than 28 days or being younger than 44 weeks of conception. Infants were classified as children aged less than 365 days. Intervention was video laryngoscopy. Comparator was direct laryngoscopy. The outcomes assessed included either intubation success rate or time to intubation. Study design was a randomized controlled parallel group trial.

We excluded studies that compared different video laryngoscopes. Furthermore, non-English written reports were excluded.

### Outcomes

Our main outcomes were first-pass intubation success rate and time to intubation. Secondary outcomes were adverse events as reported and defined by the included studies.

### Data extraction

One author extracted the data, and another author validated the extracted in order to reduce potential extraction errors. We extracted the following information from each study: authors, journal, study period, country, video laryngoscope brand, comparator, patient characteristics, intubating physicians’ experience, outcomes, funding, and potential competing interests. Extraction was performed to a pre-designed Excel spreadsheet.

### Risk of bias and evidence certainty

Risk of bias was assessed according to the Cochrane risk of bias 2.0 tool [17]. Risk of bias figures were created by using Robvis shinyapp [18]. Risk of bias was classified per outcome, and as the outcomes in our review were similar in terms of subjectivity in the outcome assessor assessment, a single risk of bias figure was enough to present the risk of bias for all outcomes. We rated the evidence certainty according to the GRADE (Grading of Recommendations Assessment, Development and Evaluation) framework [19]. In the GRADE rating, we did not downgrade due to imprecision automatically, if a confidence interval overlapped one, and instead utilized a minimally contextualized approach [20].

### Statistics

This review has been conducted according to the Cochrane handbook guidelines [21]. Analyses were performed by RevMan 5.4.1 software. In the statistical synthesis, the studies were pooled by using random effects inverse variance meta-analysis. We used risk ratios with 95% confidence intervals for dichotomous outcomes, and for rare (event rate < 10%) dichotomous outcomes, we used Peto odds ratios (OR). For continuous outcomes, we used mean difference, as all the studies used the same scale (seconds) in the assessment. If a study presented median + interquartile range, it was converted to mean and standard deviation. We assessed statistical heterogeneity by examining the *I*^*2*^ value, but it did not guide the random-effects mode choice. We aimed to reduce the heterogeneity by conducting subgroup analyses, by dividing the analysis to study setting (operation room vs NICU), weight (under or more than 5 kg), and age (neonates vs infants). A sensitivity analysis where studies with high risk of bias were eliminated was performed. Furthermore, we planned to estimate publication bias visually from a funnel plot, if at least ten studies were included [22].

This study has been reported according to the Preferred Reporting Items in Systematic Reviews and Meta-analyses (PRISMA) 2020 guidelines, and the checklist can be found in the supplementary materials.

### Protocol registration

Protocol for this review was registered in Prospero.

## Results

### Search results

**Fig. 1:**
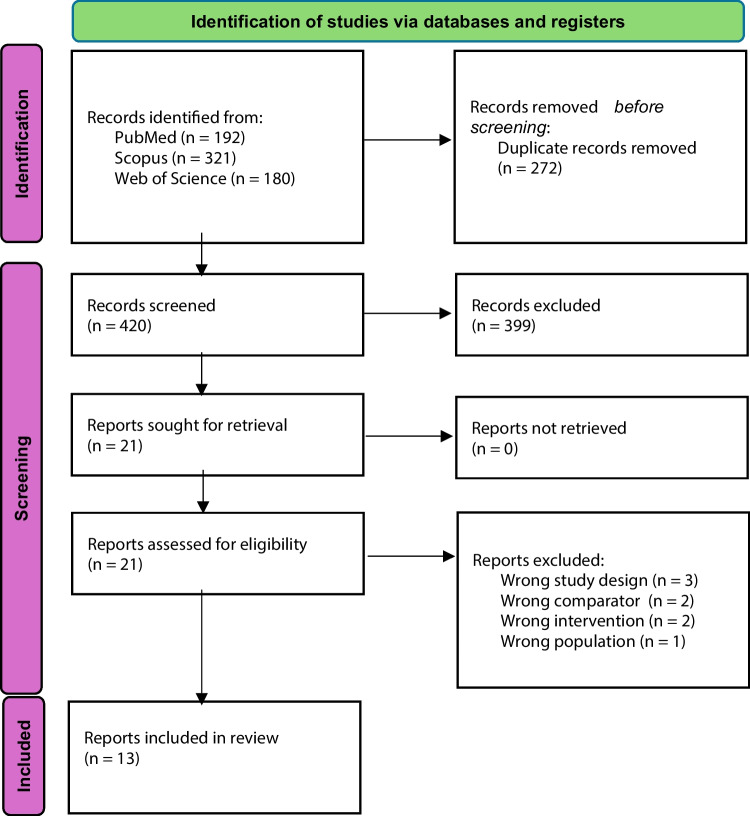
PRISMA flowchart of the study selection process

A total of 420 results were screened, 21 studies further assessed, and finally 13 randomized studies [7, 9, 23–33] with 1721 patients included (Fig. 1). Exclusions were made due to wrong study design, wrong comparator, wrong intervention, or wrong population (Fig. 1).

### Characteristics of the included studies

All of the included studies were conducted after 2010 in Asia (5 studies), the USA (3 studies), Europe (3 studies), Australia (2 studies), and Africa (1 study) (Supplementary Table 1). Four studies were conducted in neonatal intensive care units and nine studies in operation rooms. Seven studies included only neonates, and six studies also included infants. Eleven studies used back-lying intubation position, and two studies were conducted in side-laying lateral position. The intubating physician experiences had notable variation between the studies. Most studies used the C-MAC video laryngoscope and compared it to Miller direct laryngoscope (Table 1). Characteristics of the included patients are described in terms of age and weight in supplementary Table 2.

**Table 1:** Characteristics of the included studies

Study	Study setting	Age criteria	Intubation indication	Intubation position	Intubator experience	Intervention	Comparator	Main outcome
Chae et al. 2022	Operation room	< 12 months	General surgery under anesthesia	Back-lying	10 years of experience in pediatric anesthesia	UEscope	Miller of Macintosh	Time to successful intubation
Fiadjoe et al. 2012	Operation room	< 12 months	General surgery under anesthesia	Back-lying	Attending anesthesiologists with at least 50 infant intubations prior to study	Glidescope Cobalt	Miller	Time to successful intubation
Garcia-Marcinkiewicz et al. 2020	Operation room	< 12 months	General surgery under anesthesia	Back-lying	Varying experience (residents, fellows, and attendings)	C-MAC	Miller or Macintosh	First attempt success rate
Geraghty et al. 2024	NICU	0–28 days	Varying neonatal indications	Back-lying	Varying experience (at least 20 intubations and up to hundreds)	C-MAC	Miller	First attempt success rate
Goel et al. 2022	Operation room	0–28 days	General surgery under anesthesia	Back-lying	Not specified	C-MAC	Miller	Percentage of glottic opening score
Jain et al. 2017	Operation room	< 12 months	General surgery under anesthesia	Lateral position	At least 200 standard and 50 video intubations in children	C-MAC	Miller	Time to succesfull intubation
Manhas et al. 2023	Operation room	1–11 months	General surgery under anesthesia	Back-lying	At least 50 standard and 50 video intubations in children	C-MAC	Miller	Time to succesfull intubation
Moussa et al. 2016	NICU	Neonate	Varying neonatal indications	Back-lying	Pediatric residents	C-MAC	Miller	First attempt success rate
Riva et al. 2023	Operation room	< 12 months	Non-emergency surgical (98%) and non-surgical procedures (2%)	Back-lying	Anesthesiologists with varying experience of infant intubation (50% had more than 50 intubations and 25% had less than 10)	C-MAC	Miller	First attempt success rate
Salama et al. 2018	Operation room	0–28 days	Meningomyelocele or meningocele surgery under general anesthesia	Lateral position	Not specified	Glidescope Cobalt	Miller	Percentage of glottic opening score
Tao et al. 2019	Operation room	0–28 days	General surgery under anesthesia	Back-lying	At least 1000 prior intubations	Glidescope Cobalt	Miller	Time to succesfull intubation
Tippmann et al. 2023	NICU	Neonate	Varying neonatal indications	Back-lying	Pediatricians (70% had more than 10 intubations and rest less than 10)	Infantview	Miller, Macintosh, or Saling	First attempt success rate
Volz et al. 2018	NICU	Neonate	Varying neonatal indications	Back-lying	Pediatric residents with minimal experience	C-MAC	Miller	First attempt success rate

### Risk of bias

**Fig. 2:**
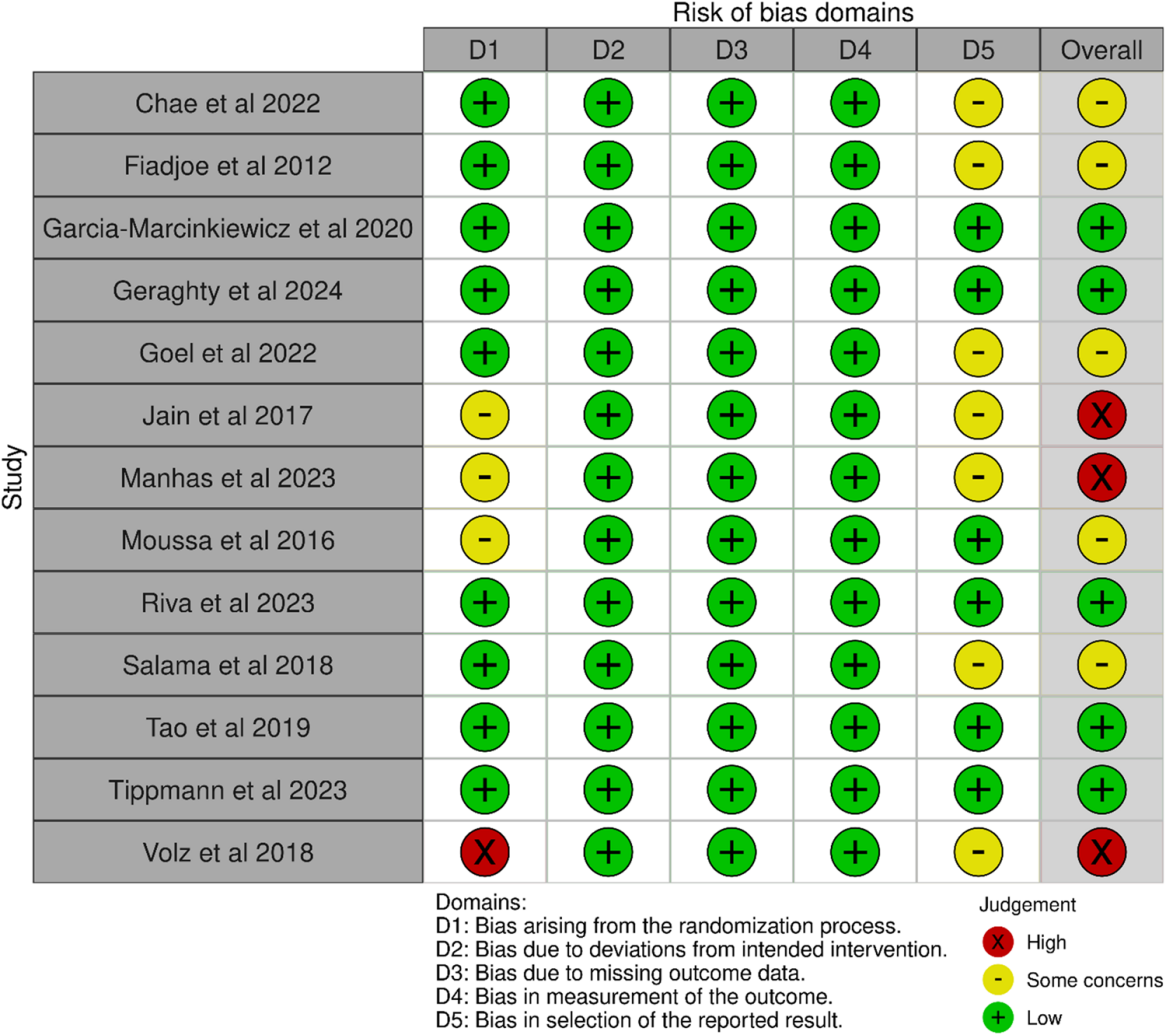
Risk of bias assessment of the included studies

Overall risk of bias was low in five, had some concerns in five, and was high in three of the studies (Fig. 2). Most issues came from improper description or conduction of randomization and selection bias in results reporting.

### Intubation success rate

**Fig. 3:**
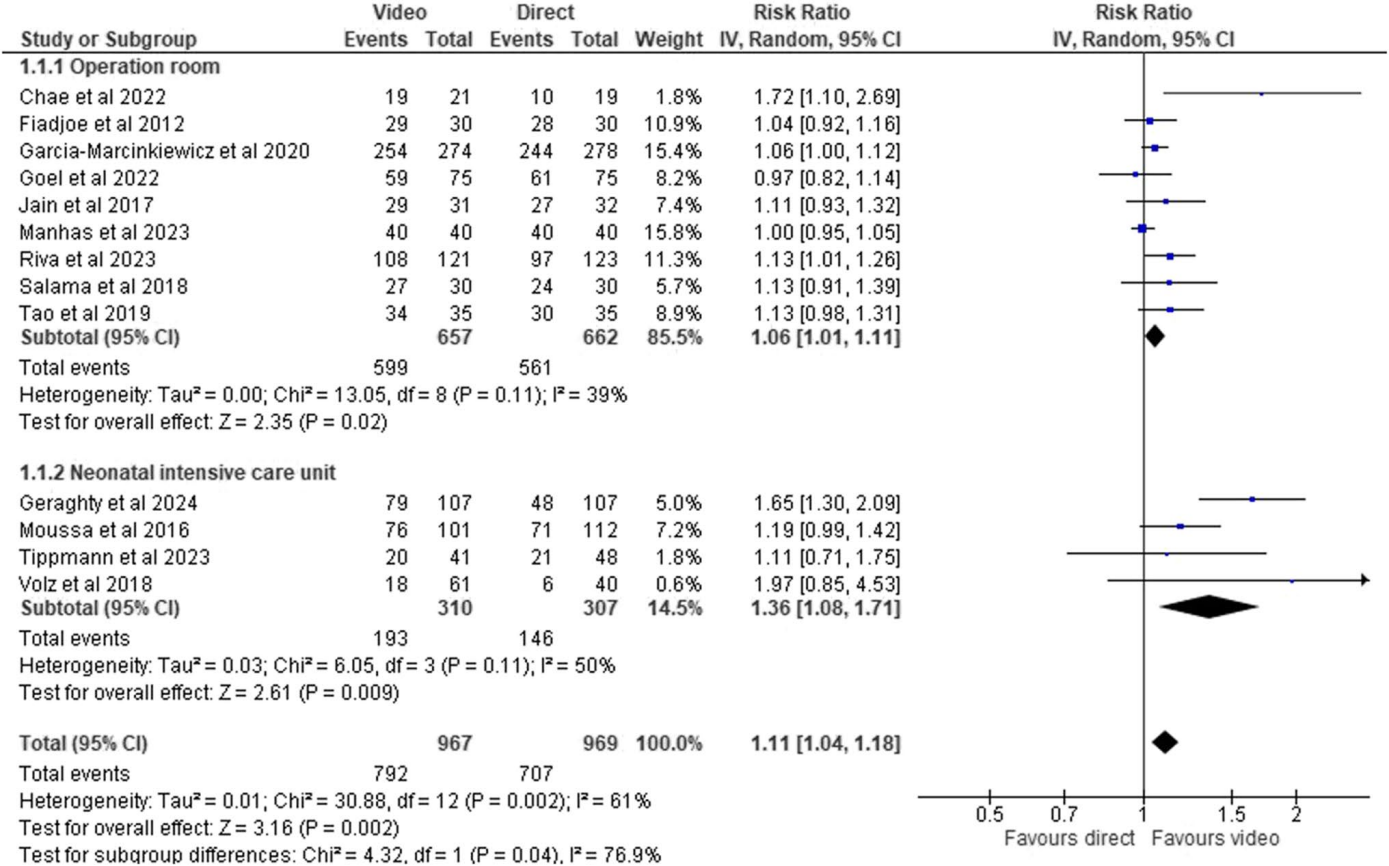
First-pass intubation success rates between video laryngoscopy and direct laryngoscopy stratified by the intubation setting (operation room and neonatal intensive care unit)

Overall, the intubation success rate was analyzed in all 13 studies (1936 patients), and the first attempt success rate was higher in the video laryngoscopy than in the direct laryngoscopy group (RR 1.11, CI 1.04–1.18) (Fig. 3). Certainty of evidence was ranked as moderate (Table 2).

**Table 2:** Evidence certainty assessed according to GRADE for main outcomes

Outcome	*N* of studies (*n* of participants)	Absolute success rate in control group	Relative risk (95% CI)	Absolute effect	GRADE
First intubation success rate	13 (1936)	73 per 100	1.11 (1.04–1.18)	8 more per 100 (3 more to 13 more)	Moderate*
Operation room	9 (1319)	85 per 100	1.06 (1.01–1.11)	5 more per 100 (1 more to 9 more)	Moderate*
NICU	4 (617)	48 per 100	1.36 (1.08–1.71)	17 more per 100 (4 more to 34 more)	Moderate*
Neonate	7 (897)	68 per 100	1.18 (1.03–1.36)	12 more per 100 (2 more to 24 more)	Moderate*
Infants	6 (1039)	85 per 100	1.06 (1.00–1.20)	5 more per 100 (0 more to 16 more)	Moderate*
Weight < 5 kg	8 (1141)	63 per 100	1.17 (1.05–1.30)	11 more per 100 (3 more to 19 more)	Moderate*
Weight 5 kg or more	5 (795)	89 per 100	1.05 (0.98–1.11)	4 more per 100 (2 less to 9 more)	Moderate*
Back-lying position	11 (1813)	72 per 100	1.11 (1.03–1.19)	8 more per 100 (2 more to 14 more)	Moderate*
Lateral position	2 (123)	83 per 100	1.12 (0.97–1.28)	10 more (2 less to 22 more)	Low**
Time to intubation	11 (1279)	Not applicable	Not applicable	1.2 s more (2.2 s less to 4.6 s more)	Low**
Adverse events			Odds ratios		
Desaturation rate	8 (1312)	14 per 100	0.62 (0.42–0.93)	5 less per 100 (8 less to 1 less)	Moderate*
Intubation trauma	8 (11,180)	2 per 100	0.24 (0.07–0.85)	1 less per 100 (2 less to 0 less)	Low**
Bronchospasm	3 (944)	1 per 100	0.52 (0.09–3.01)	Not applicable	Very low***

*Downgraded due to risk of bias

**Downgraded due to risk of bias and imprecision

***Downgraded due to risk of bias and twice due to imprecision

In the subgroup analyses, the first attempt success rates were higher in the video laryngoscopy group in both operation rooms (RR 1.06, CI 1.01–1.11; 9 studies) and neonatal intensive care units (RR 1.36, CI 1.08–1.71; 4 studies; Fig. 3). Certainty of evidence was moderate (Table 2). Seven studies were conducted in neonates (age < 28 days), and six studies also included infants. The first attempt success rates were higher in the video laryngoscopy group in neonates (RR 1.18, CI 1.03–1.36) and in infants (RR 1.06, CI 1.00–1.20; moderate certainty of evidence) (Fig. S1, Table 2). In a weight-stratified analysis, the success rate with video laryngoscope was higher among neonates and infants weighing less than 5 kg (RR 1.17, CI 1.05–1.30; 8 studies), but then no evidence of a difference between video and direct laryngoscopy was seen among those weighing 5 kg or more (RR 1.05, CI 0.98–1.11; 5 studies; Fig. S2). Certainty of evidence certainty was moderate (Table 2). Two studies utilized lateral intubation position due to anesthesia indication (congenital repair of spinal malformations), and the first-pass intubation success rate seemed to favor the video laryngoscope group (RR 1.12, CI 0.97–1.28). The intubation success rate was higher in the video laryngoscope group in the standard back-lying intubation position (RR 1.11, CI 1.03–1.19; 11 studies; Fig. S3). Certainty of evidence was moderate (Table 2). The studies did not show a sign of publication bias in the funnel plot (Fig. S4). Furthermore, we conducted a sensitivity analysis where studies with a high risk of bias were excluded and the effect estimates did not change (Fig. S5).

### Time to intubation

**Fig. 4:**
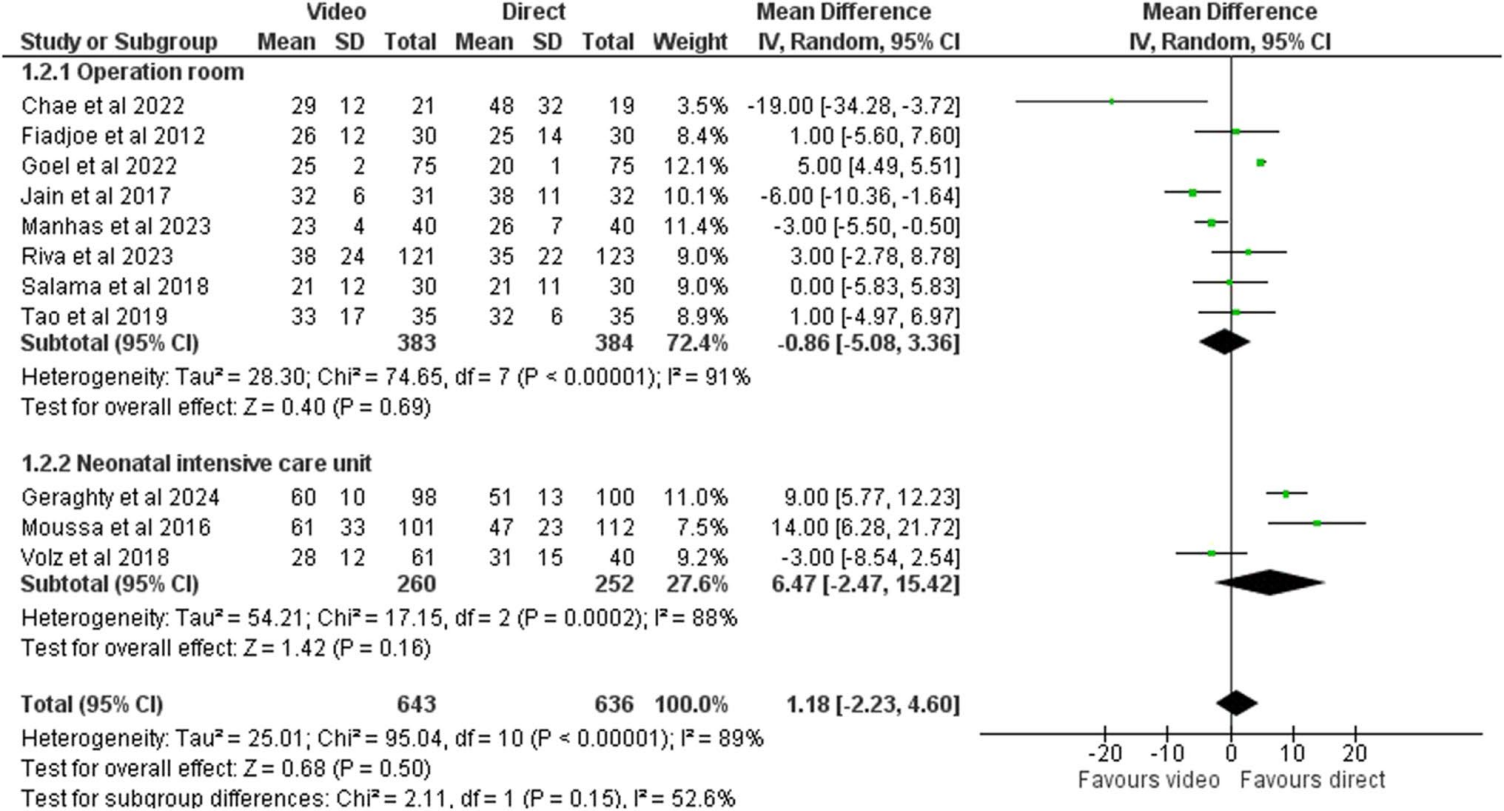
Time to successful intubation stratified by the study setting

Time to intubation was assessed in ten studies. The mean time difference between video laryngoscopy and direct laryngoscopy group was 1.2 s (CI − 2.2 to 4.6 s; Fig. 4). Certainty of evidence was low (Table 2). The mean difference in the time between video and direct laryngoscopy was − 0.9 s (− 5.1 to 3.4) in the operation room and 6.5 s (− 2.5 to 15.4) in the NICU. We did not detect signs of publication bias (Fig. S6). The overall outcome estimate did not change for the operation room in a sensitivity analysis where studies with a high risk of bias were excluded, but the time to intubation was longer in the NICU setting (Fig. S7).

### Adverse events

Desaturation during intubation was assessed in eight studies, and the rate was lower in the video laryngoscopy group (OR 0.62, CI 0.42–0.93; Fig. S8). The evidence certainty was ranked as moderate (Table 2). Intubation-related trauma was rare in both groups, and it was lower in the video laryngoscopy group (OR 0.24, CI 0.07–0.85; Fig. S8). Certainty of evidence was low (Table 2). Bronchospasm was assessed in three studies, and the effect estimate had high uncertainty (OR 0.52, CI 0.09–3.01; Fig. S8). Certainty of evidence was very low (Table 2). We did not detect signs of publication bias in the funnel plot (Fig. S9). A sensitivity analysis without studies with a high risk of bias did not change the effect estimates (Fig. S10).

## Discussion

This systematic review and meta-analysis of 13 randomized trials found moderate certainty evidence that first-pass endotracheal intubation success rate was higher both in neonates and infants when video laryngoscopy was used instead of direct laryngoscopy. In particular, this improvement was seen in neonates and infants weighing less than 5 kg and in studies conducted in neonatal intensive care units. Time to intubation did not differ between video and direct laryngoscopy, and the adverse event rates were comparable between the groups.

Understanding the anatomy of the neonatal airway is crucial during intubation to prevent pharyngeal blockage and accurately evaluate the larynx. Unique characteristics of the neonatal airway, observable during laryngoscopy, differ from those in older children. Neonates predominantly breathe through their noses and may struggle to respire through the mouth when nasal passages are obstructed. The tongue in neonates is comparatively large, and the epiglottis exhibits characteristics of being larger, longer, less flexible, and narrower. The larynx occupies a higher and more anterior position in relaxation to the cervical vertebrae, reaching the typical “adult position” around the age of six. Notably, the neonatal airway assumes a funnel shape, with the narrowest segment at the subglottic space, unlike in older patients where the glottis holds this distinction. Due to the absence of posterior tracheal cartilage, the neonatal airway is more susceptible to inspiratory collapse and obstruction [34, 35]. Thus, these factors undermine the importance and difficulty of neonatal and infant intubation.

As the guidelines of medical management in terms of meconium suction [36] and surfactant administration have changed to less invasive [37], the number of endotracheal intubations has decreased in neonatology. In addition, to minimize associated morbidities, the most recent resuscitation protocols highlight the preference for noninvasive respiratory support in the management of all respiratory disorders in spontaneously breathing preterm infants [38]. Consequently, both pediatricians and neonatologist have fewer intubations now than before [11, 12]. The addition of laryngeal mask airway to resuscitation protocols and for surfactant delivery, in the recent guideline updates, will further reduce the intubation numbers [39, 40]. Therefore, as intubations become less frequent, the intubation skills and routine of physicians are at risk. Thus, it is vital to maintain the intubation practices as safe as possible for the smallest patients. Based on the results of this meta-analysis, the use of video laryngoscopy improves first-pass intubation success rates in neonatal intensive care units. This is in line with a recent Cochrane meta-analysis which focused only on neonates [6]. It is well known that intubation experience correlates with the success rate in small children [41], and thus it was unsurprising that the intubation success rates were higher in the operation room and in bigger infants in both video and direct laryngoscopy groups. However, for anesthesiologists, it is important to be able to perform well both with video laryngoscopy and with direct laryngoscopy, as in emergent situations video laryngoscopy may not always be available [4]. Another positive side in the use of video laryngoscopy is that the instructor can more easily teach intubation to less experienced providers and thus improve the learning curve for intubation. This has been demonstrated in previous randomized trials [16, 28, 32]. However, it must be noted that a practice that prioritizes video laryngoscopy will probably diminish the already dwindling skills of direct laryngoscopy increasing the technology dependency in neonate airway management. Also, in a global scale, a large part of neonatal intensive care units do not have access to video laryngoscopy.

Time to intubation did not differ between the groups. Once familiar, the two techniques do not differ greatly in terms of hand technique. The included trials showed that the odds for intubation trauma and desaturation rates were lower in the video laryngoscope group. The studies were too small to detect differences in rare adverse events, such as bronchospasm.

### Implications for future research or practice

Based on the results of this review, the use of a video laryngoscope should be the first line choice in neonate intubation and in low-weighing infants, as it improves the success rates most in these groups. However, the first-pass success rate in infants in the operation room was relatively high in the direct laryngoscopy group when anesthesiologists performed the intubation, and thus video laryngoscopy is rather a choice of additional value in safety and teaching.

Future research is especially needed from neonatal intensive care units, as the first-pass success rates there are lower than in a controlled operation room environment. Although video laryngoscopy improved the intubation success rates, critical care may necessitate the use of additional aids (such as the use of a bougie). Increasing the safety of neonatal peri-intubation period should be the focus of intervention studies. As an example, a recent Australian study found the use of high-flow nasal cannula during the intubation to improve the success rates notably by improving the neonates physiological stability during the intubation [42]. Furthermore, future studies should aim to analyze, for example, the use of video laryngoscopy in surfactant administration when it is done using minimal invasive technique.

### Strengths and limitations

The main strength compared to previous reviews was that we included both neonates and infants and performed subgroup analyses based on the weight alongside the typical age stratification used.

The main limitations were the heterogeneity in terms of intubation performer experience, which we were unable to adjust in our analysis. For adverse event outcomes, the studies were too small to detect meaningful differences. Furthermore, we excluded non-English written studies, which may have left potential studies out of this review and further decreases the generalizability of our results.

## Conclusion

We found moderate quality evidence that the use of video laryngoscopy improves first attempt success rates in neonate and infant intubations, while the time to intubation did not differ between video and direct laryngoscopy groups. Further studies are still needed to improve the first intubation success rates in neonates in neonatal intensive care unit setting, as these were below the success rates of infants and operation room.

## Supplementary Information

Below is the link to the electronic supplementary material.

Supplementary file1 (DOCX 186 KB)

## Data Availability

All data not found from the manuscript or supplementary materials that were generated during the review process are available upon request from the corresponding authors.

## Declarations

### Protocol registration

PROSPERO 2023 CRD42023492861 Available from https://www.crd.york.ac.uk/prospero/display_record.php?ID=CRD42023492861
