# Supplementary material for: Video laryngoscopy in neonate and infant intubation—a systematic review and meta-analysis
Source: Eur J Pediatr. 2024 Nov 20;184(1):34. doi: 10.1007/s00431-024-05839-2 (PMC11579204; doi:10.1007/s00431-024-05839-2)
Supplement: Supplementary file 1 — Supplementary file1 (DOCX 186 KB) [file 431_2024_5839_MOESM1_ESM.docx]

**Supplementary materials**

**Search strategy**

**Supplementary Table 1** Characteristics of the included studies.

**Supplementary Table 2** Characteristics of the patients in the included studies.

**Figure S1** First attempt intubation success rate between video laryngoscopy and direct laryngoscopy stratified by the age of the children (neonates vs infants).

**Figure S2** First attempt intubation success rate between video laryngoscopy and direct laryngoscopy stratified by the weight (less than 5 kg vs 5 kg or more).

**Figure S3** First attempt intubation success rate between video laryngoscopy and direct laryngoscopy stratified by the intubation position.

**Figure S4** Funnel plot to evaluate the possibility of publication bias in the outcome of first attempt success rate:

**Figure S5** Sensitivity analysis for the first intubation success rate. Studies with high risk of bias were excluded.

**Figure S6** Funnel plot to evaluate the possibility of publication bias in the outcome of time to intubation.

**Figure S7** Sensitivity analysis for the time to intubation. Studies with high risk of bias were excluded.

**Figure S8** Adverse events related to intubation between video laryngoscopy and direct laryngoscopy.

**Figure S9:** Funnel plot to evaluate the possibility of publication bias in the outcome of adverse events.

**Figure S10:** Sensitivity analysis for the adverse event outcome. Studies with high risk of bias were excluded.

**2**

**3**

**4**

**5**

**6

7**

**8**

**9

10**

**11**

**12**

**13**

**14**

**Complete search strategy:**

Pubmed: Search: Video AND (laryngoscope or laryngoscopy) AND (infant or newborn or neonate)

("video s"[All Fields] OR "videoed"[All Fields] OR "videotape recording"[MeSH Terms] OR ("videotape"[All Fields] AND "recording"[All Fields]) OR "videotape recording"[All Fields] OR "video"[All Fields] OR "videos"[All Fields]) AND ("laryngoscope s"[All Fields] OR "laryngoscopes"[MeSH Terms] OR "laryngoscopes"[All Fields] OR "laryngoscope"[All Fields] OR "laryngoscopic"[All Fields] OR "laryngoscopical"[All Fields] OR "laryngoscopically"[All Fields] OR ("laryngoscopy"[MeSH Terms] OR "laryngoscopy"[All Fields] OR "laryngoscopies"[All Fields])) AND ("infant"[MeSH Terms] OR "infant"[All Fields] OR "infants"[All Fields] OR "infant s"[All Fields] OR ("infant, newborn"[MeSH Terms] OR ("infant"[All Fields] AND "newborn"[All Fields]) OR "newborn infant"[All Fields] OR "newborn"[All Fields] OR "newborns"[All Fields] OR "newborn s"[All Fields]) OR ("infant, newborn"[MeSH Terms] OR ("infant"[All Fields] AND "newborn"[All Fields]) OR "newborn infant"[All Fields] OR "neonatal"[All Fields] OR "neonate"[All Fields] OR "neonates"[All Fields] OR "neonatality"[All Fields] OR "neonatals"[All Fields] OR "neonate s"[All Fields]))

SCOPUS: Video AND (laryngoscope or laryngoscopy) AND (infant or newborn or neonate)

Web Of Science: Video AND (laryngoscope or laryngoscopy) AND (infant or newborn or neonate)

**Supplementary Table 1** Characteristics of the included studies.

| Study | Country | Study period | Funding | COI |
| --- | --- | --- | --- | --- |
| Chae et al 2022 | South-Korea | 2020-2021 | Reported | None to report |
| Fiadjoe et al 2012 | USA | Not reported | Not reported | Not reported |
| Garcia-Marcinkiewicz et al 2020 | USA and Australia | 2018-2019 | Reported (company funding) | Reported |
| Geraghty et al 2024 | Ireland | 2021-2023 | Reported | Reported |
| Goel et al 2022 | India | Not reported | None to report | None to report |
| Jain et al 2017 | India | Not reported | Not reported | None to report |
| Manhas et al 2023 | India | 2020-2022 | None to report | None to report |
| Moussa et al 2016 | Canada | 2011-2013 | Reported | None to report |
| Riva et al 2023 | Australia, Canada, Italy, Switzerland, USA | 2020-2022 | Reported | Reported |
| Salama et al 2018 | Egypt | 2016-2018 | None to report | None to report |
| Tao et al 2019 | China | Not reported | None to report | None to report |
| Tippmann et al 2023 | Germany | 2020-2021 | None to report | None to report |
| Volz et al 2018 | USA | 2014-2015 | Not reported | None to report |

**Supplementary Table 2** Characteristics of the patients in the included studies.

| Study | Age | | Weight | |
| --- | --- | --- | --- | --- |
|  | Video | Direct | Video | Direct |
| Chae et al 2022 | Median 72 days | Median 84 days | Median 4.3 kg | Median 5.5 kg |
| Fiadjoe et al 2012 | Mean 5.9 months | Mean 5.1 months | N/A | N/A |
| Garcia-Marcinkiewicz et al 2020 | Mean 5.6 monts | Mean 5.4 months | Mean 6.5 kg | Mean 6.6 kg |
| Geraghty et al 2024 | Gestational age median 30 weeks | Gestational age median 28 weeks | Median 1.3 kg | Median 1.3 kg |
| Goel et al 2022 | Mean 5.2 days | Mean 4.7 days | Mean 2.4 kg | Mean 2.3 kg |
| Jain et al 2017 | Median 4.8 months | Median 4.0 months | Median 5.6 kg | Median 5.5 kg |
| Manhas et al 2023 | Mean 6.6 months | Mean 7.3 months | Mean 6.4 kg | Mean 6.4 kg |
| Moussa et al 2016 | Gestational age median 29 weeks | Gestational age median 29 weeks | Median 1.4 kg | Median 1.6 kg |
| Riva et al 2023 | Median postmenstrual age 46 weeks | Median postmenstrual 44 weeks | Mean 4.4 kg | Mean 4.2 kg |
| Salama et al 2018 | Mean 3.9 days | Mean 4.1 days | Mean 3.2 kg | Mean 3.4 kg |
| Tao et al 2019 | Mean 9.2 days | Mean 10.8 days | Mean 3.5 kg | Mean 3.6 kg |
| Tippmann et al 2023 | Gestational age median 29.0 weeks | Gestational age median 29.0 weeks | Median 1.2 kg | Median 1.1 kg |
| Volz et al 2018 | Gestational age mean 33 weeks | Gestational age mean 32 weeks | Mean 1.9 kg | Mean 1.8 kg |

**Figure S1:** First attempt intubation success rate between video laryngoscopy and direct laryngoscopy stratified by the age of the children (neonates vs infants).


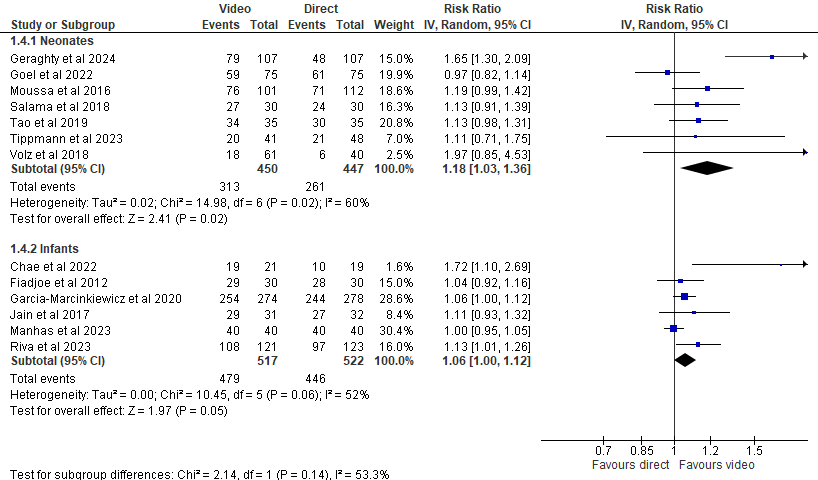


**Figure S2** First attempt intubation success rate between video laryngoscopy and direct laryngoscopy stratified by the weight (less than 5 kg vs 5 kg or more).


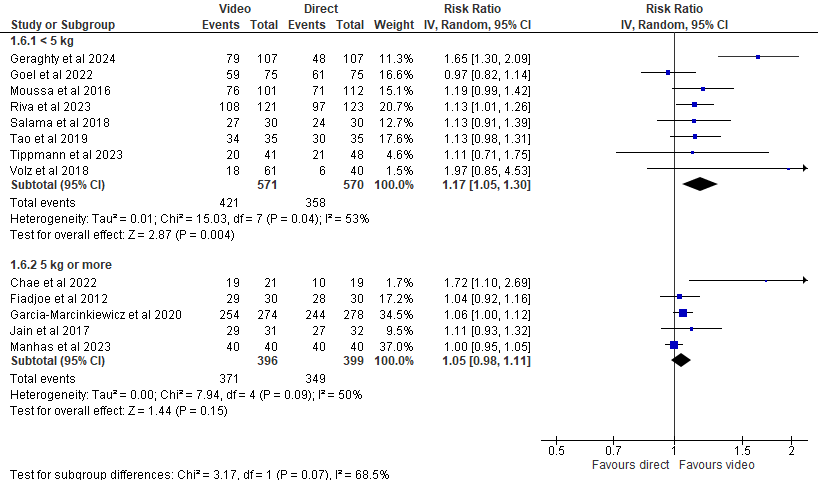


**Figure S3** First attempt intubation success rate between video laryngoscopy and direct laryngoscopy stratified by the intubation position.


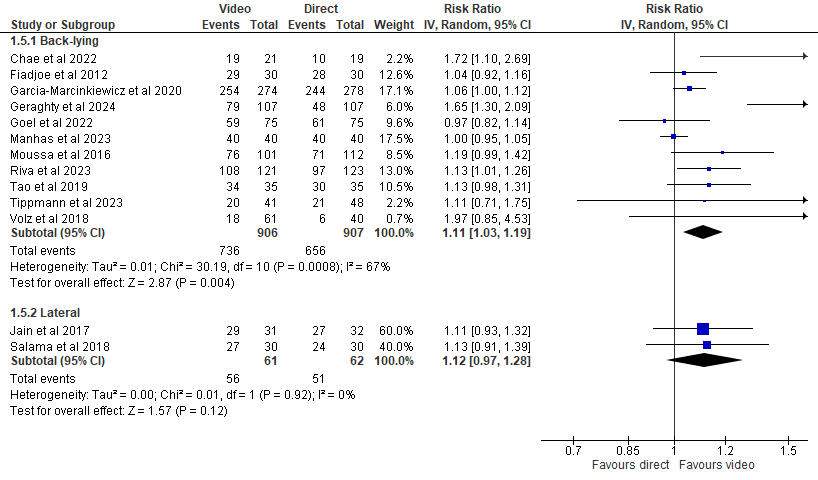


**Figure S4** Funnel plot to evaluate the possibility of publication bias in the outcome of first attempt success rate:


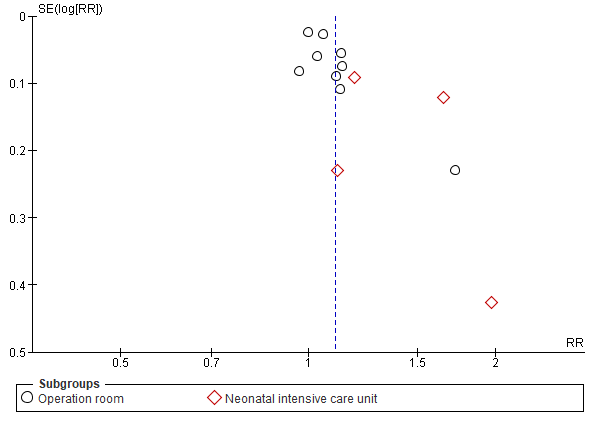


**Figure S5** Sensitivity analysis for the first intubation success rate. Studies with high risk of bias were excluded.


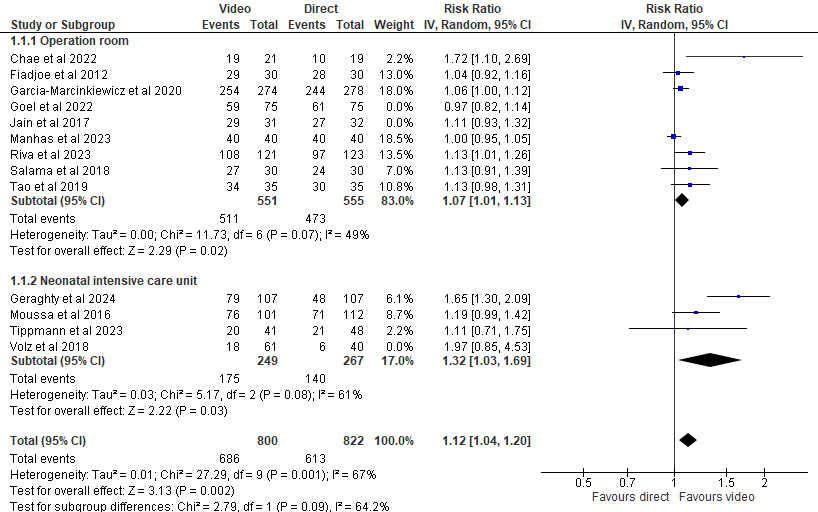


**Figure S6** Funnel plot to evaluate the possibility of publication bias in the outcome of time to intubation.


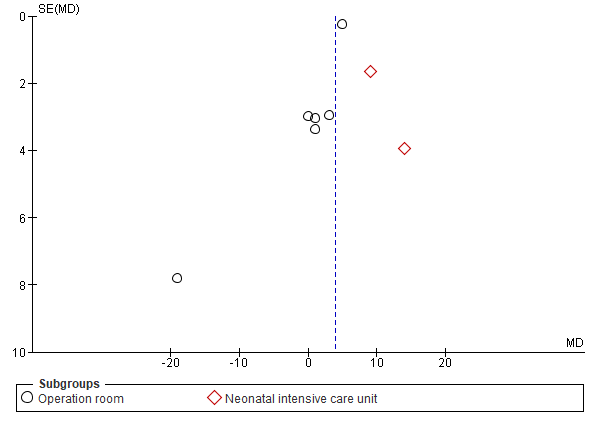


**Figure S7** Sensitivity analysis for the time to intubation. Studies with high risk of bias were excluded.


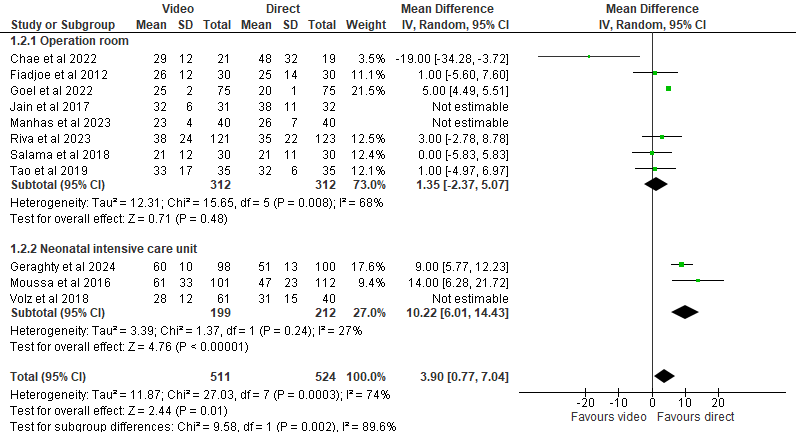


**Figure S8** Adverse events related to intubation between video laryngoscopy and direct laryngoscopy.


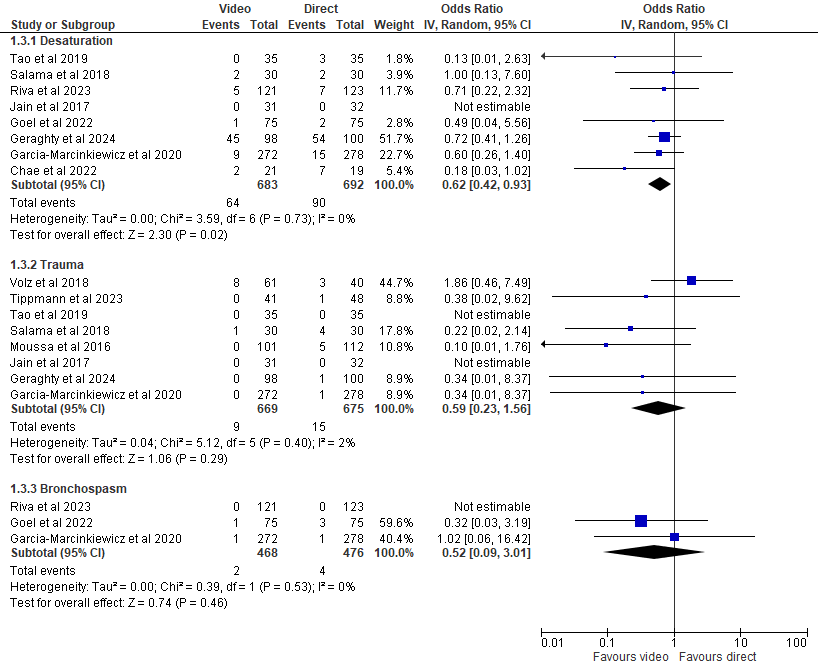


**Figure S9:** Funnel plot to evaluate the possibility of publication bias in the outcome of adverse events.


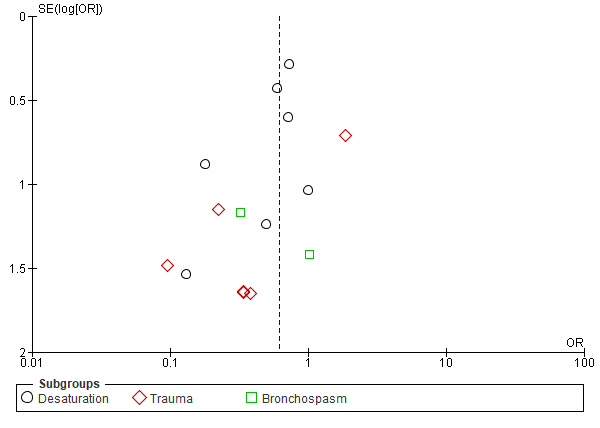


**Figure S10** Sensitivity analysis for the adverse event outcome. Studies with high risk of bias were excluded.

**
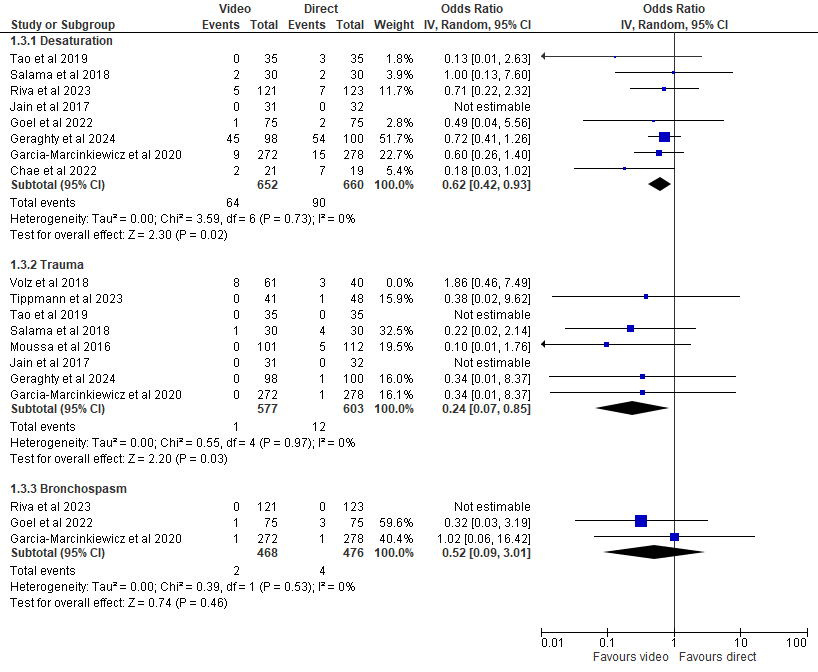
**
